# Isolation, molecular typing and antimicrobial resistance of *Clostridium difficile* in dogs and cats in Lanzhou city of Northwest China

**DOI:** 10.3389/fvets.2022.1032945

**Published:** 2022-11-18

**Authors:** Gui-Lin Wen, Shi-Hong Li, Zhe Qin, Ya-Jun Yang, Li-Xia Bai, Wen-Bo Ge, Xi-Wang Liu, Jian-Yong Li

**Affiliations:** Key Lab of New Animal Drug Project of Gansu Province, Key Lab of Veterinary Pharmaceutical Development of Ministry of Agriculture and Rural Affairs, Lanzhou Institute of Husbandry and Pharmaceutical Sciences of Chinese Academy of Agricultural Sciences, Lanzhou, China

**Keywords:** *Clostridium difficile*, dog, cat, molecular typing, antimicrobial resistance

## Abstract

*Clostridium difficile* infection (CDI) in human and animals belonged usually to antibiotic-associated diarrhea, ranging in severity from mild to life-threatening intestinal tract illnesses. This study aimed to isolation and characterization, toxin genes test, molecular typing, and drug sensitivity of *Clostridium difficile* (*C. difficile*) which were isolated from clinical diseased dogs and cats. A total of 247 clinical samples were collected from five animal hospitals in Lanzhou City of Northwest China, of which dogs and cats accounted for 74.9% (185/247) and 25.1% (62/247), respectively. We successfully identified 24 *C. difficile* strains by 16S rRNA and Matrix-Assisted Laser Desorption/Ionization Time of Fight Mass Spectroscopy (MALDI-TOF-MS). 10.3% (19/185) of dogs and 8.1% (5/62) of cats were positive for *C. difficile*. Among them, 16 strains were toxic and 8 were non-toxic, with a toxic rate of 57.9% (11/19) in dogs and 100% (5/5) in cats. A total of 10 STs and 10 RTs were identified in this study. The percentages of ST42 (RT106) and ST2 (RT014/LW01) among 16 toxic strains were 41.7 and 12.5%, respectively. However, ST3 (RT001), ST1 (RT027), ST133 (LW04), and ST-UN (LW04) had only one strain. ST42 (RT106) was the most common genotype and RT027 strain was first isolated in China from pets. Antimicrobial susceptibility test showed that isolates were extremely sensitive to vancomycin and metronidazole but were resistant to erythromycin and ciprofloxacin. The drug resistant rates to clindamycin, levofloxacin, moxifloxacin and meropenem were 62.5, 20.8, 16.7, and 8.3%, respectively. In conclusion, *C. difficile* was quietly prevalent in dogs and cats in Lanzhou city with RT106 and RT014 as the main ribotypes. The CDI in pets should be paying more attention and further studies are needed.

## Introduction

*C. difficile* is an obligate anaerobic spore-forming Gram-positive opportunistic pathogen isolated first from infants by Hall in 1935 (1). The toxin-producing bacteria mainly colonize in the intestinal tract of human and animals (2). However, *C. difficile* spores are widely distributed in the environment, including soil, water, plants and animals, particularly in hospital facilities (3).

*C. difficile* pathogenicity is primarily mediated by its toxin. Toxin-producing *C. difficile* proliferates in the intestine and releases toxins that cause *C. difficile* infection (CDI) in the intestinal mucosa, such as diarrhea, colitis, and even death in severe cases. *C. difficile* can produce a 304 kDa enterotoxin A (TcdA), a 270 kDa cytotoxin B (TcdB), and a *C. difficile* binary toxin (CDT) in the intestine (4–6). The genes encoding the toxins A and B are located in the same pathogenicity locus (PaLoc) of the chromosome (6, 7). This region also contains three additional accessory genes as the positively regulated gene *tcdR*, the negatively regulated gene *tcdC*, and the porin gene *tcdE* (5). Toxin A and toxin B have similar pathogenic mechanisms in that both of them can inactivate the Rho protein family in host cells *via* glycosylation modification (6). They cause intestinal cytoskeleton loss and structural disruption, resulting in strong inflammatory responses and multiple clinical symptoms, such as enteritis and diarrhea (7). In addition, some strains produce binary toxins encoded by *cdtA* and *cdtB* genes located outside the PaLoc. The binary toxins act as an ADP ribosyltransferase and break down action on the cell surface, causing intestinal epithelial cell variation and apoptosis (8). However, the pathogenic mechanism of CDT remains unknown (9).

The overall incidence and number of severe cases of CDI have increased recently due to the prevalence of highly virulent strains and the increasing rate of community-acquired infections (10). Based on molecular typing, CDI outbreaks in Europe and the United States have been linked to large-scale epidemics of highly virulent strains RT027 and RT078 (11). Nevertheless, CDI and CDI-related epidemics are not limited to these ribotypes (RTs). RTs 001, 002 and 014/020 are frequently associated with CDI clusters in the United States and Europe (12, 13). Recent study revealed that the isolation rate of *C. difficile* from hospitalized patients ranged from 9% to 14% in China (14, 15). The main epidemic strains were ST37(RT017), ST3(RT001), ST54(RT012) (15–18).

Community-acquired CDI is one of the main factors for the increased incidence of CDI. With the progress in research on community-acquired CDI, more cases of animals carrying *C. difficile* have been reported (19). The most common RTs isolated from dogs in Spain are RT106 and RT154 (20). In addition, virulent strains RT027 and RT078 are isolated in dogs in Canada and diarrheic calves in Germany (21, 22). Whole-genome sequencing revealed that *C. difficile* isolates causing CDI between animal and human were genetically related (23). Since companion animals such as dogs and cats live in close proximity with humans, susceptible people may become carriers of *C. difficile* when exposed to dogs and cats with CDI (24).

Antibiotics such as vancomycin, metronidazole, and fidaxomicin are the mainstays for CDI treatment with high cure rate (25). However, the high recurrence of CDI treated with these antibiotics cannot be neglected. The mortality and treatment costs of recurrent CDI was considerably high (26). On the other hand, *C. difficile* drug resistance is also increasing, leading to clinical CDI treatment failure and promoting CDI's occurrence and recurrence, thus bringing great difficulties to the treatment, prevention, and control of clinical CDI (27).

In recent years, with the rapid increase in pets and pet hospitals in China, the exposure of dogs and cats to individuals susceptible to CDI and environments contaminated with *C. difficile* and the use of antibiotics has led to increasingly severe risk factors for *C. difficile* colonization and transmission about CDI epidemiology on pets. However, there has been few studies in China on the clinical carriage of *C. difficile* in pets. Therefore, this study investigated the carriage of *C. difficile* in hospitalized dogs and cats using molecular epidemiology and resistance mechanisms to prevent large-scale transmission of *C. difficile*.

## Materials and methods

### Sample collection

Between March 2021 and December 2021, a total of 247 samples with anal swab were collected from dogs (*n* = 185) and cats (*n* = 62). There are five animal hospitals in Lanzhou City, Gansu Province: Qilihe District, Anning District, Xigu District, and Chengguan District. Five animal hospitals (denoted by “A,” “B,” “C,” “D,” “E”), “A” is a general practice hospital in Qilihe District, “B” and “C” are referred to the central referral hospital in Qilihe District and Anning District, and “D” and “E” are community hospitals in Chengguan District and Xigu District. “A” is the largest animal hospital with the most cases in Lanzhou City. “B” and “C,” as the referral center hospital, have a relatively small number of cases and treat severe diseases. “D” and “E”, as a community hospital, have a relatively small number of cases and treat mainly animals with vaccination and common diseases. All samples consisted of “A” (48.2%, 119), “B” (13.4%, 33), “C” (4.0%, 10), “D” (18.6%, 46), and “E” (15.8%, 39). In addition, all procedures were conducted following an approved protocol by the Institutional Animal Care and Use Committee at the Lanzhou Institute of Husbandry and Pharmaceutical Sciences of the Chinese Academy of Agricultural Sciences.

### Bacterial isolation and identification

Samples were collected with a disposable sampling anal swab from five animal hospitals in downtown Lanzhou city and inserted into Amies transport medium (Qingdao Haibo Biotechnology Co., Ltd, Qingdao, China). Samples were kept at 4°C before being sent to the laboratory within 12 h. Subsequently, samples were inoculated into a 5 mL cycloserine-cefoxitin fructose agar (CCFA) medium (an enriched, selective, and differential medium for the isolation of *C. difficile*) and placed in a Forma Anaerobic System (Thermo Scientific, USA) (28). The pretreated samples were incubated anaerobically (85% N_2_, 10% H_2_, and 5% CO_2_) at 37 °C for 7 d. Then 10 μL of the enriched culture was inoculated onto Chrome ID *C. difficile* agar medium (Merial, France) using the four-zone streak plate cultivation method and incubated anaerobically at 37°C for 48 h. Plates with growing colonies were placed under UV light at 365 nm to observe fluorescent spots. Single blue-fluorescent colony was inoculated onto an anaerobic agar medium (Beijing Land Bridge Technology Co., Ltd, China). Finally, the single colony morphology was observed and stained by Gram's method after 48 h incubation.

The isolates were identified using a matrix-assisted laser desorption–ionization time-of-flight mass spectrometry system (MALDI*-*TOF MS) (Bruker, Germany) (30). Briefly, colonies with blue fluorescence (a characteristic of *C. difficile*) and Gram-positive staining on the chromogenic medium were re-cultured for 48 h. A small number of fresh colonies were then evenly coated on the VITEK MS target plate. A 1 μL of α-Cyano-4-hydroxycinnamic acid solution was added dropwise to these colonies. The VITEK MS target plate was placed in MALDI–TOF-MS after the substrate had dried naturally. The mass spectral data were acquired between 2,000 and 20,000 Da. Finally, the strains were identified by comparing mass data to a database of standard *C. difficile* in data base through the Biotyper software.

### Toxin gene test and *TcdC* gene sequencing

Single colonies were inoculated into an anaerobic liquid medium and incubated anaerobically overnight. Following the operation manual procedures, genomic DNA was extracted from the bacterial solution using the Bacterial DNA Extraction Kit (Solarbio Technology Co., Ltd., Beijing, China) and stored at−20 °C. The extracted DNA was used as a template for five-fold polymerase chain reaction (PCR) amplification of the toxin genes *tcdA, tcdB*, and *CDT*, and the 16S rRNA following the method recommended by Cheng et al. (31). PCR amplification primer sequences were seen in Table 1. Additionally, A 1.8 kb at the 3' terminal of the *tcdA* gene was examined for deletion. The *tcdC* gene was also amplified and detected using the method proposed by Curry et al. (34). The sequence results were compared to the *tcdC* gene standard sequence (NC_009089.1, a gene bank accession number for tcdC gene sequence of *Clostridioides difficile* 630) to identify any gene deletion.

**Table 1:** PCR amplification primer sequences.

**Gene**	**Primer**	**Primer sequence (5′ − 3′)**	**Primer Concentration (μM)**	**Gene length (bp)**	**References**
*tcdA*	TCDA-F3345	ATAAGGCAATTCAGTGGTA	0.6	629	(32)
	TCDA-R3969	AGTTCCTCCTGCTCTATGAAATG	0.6		
*tcdB*	TCDB-F5670	CCAAARTGGAGTGTTACAAACAGGTG	0.4	410	
	TCDB-R6079A	GCATTTCTCCATTCTCAGCAAAGTA	0.2		
	TCDB-R6079B	GCATTTCTCCGTTTTCAGCAAAGTA	0.2		
*cdtA*	CDTA-F739	GGGAAGCACTATATTAAAGCAGAAGC	0.05	221	
	CDTA-F738B	GGGAAACATTATATTAAAGCAGAAGC	0.05		
	CDTA-R958	CTGGGTTAGGATTATTTACTGGACA	0.1		
*cdtB*	CTDB-F617	TTGACCCAAAGTTGATGTCTGATTG	0.1	262	
	CDTB-R878	CGGATCTCTTGCTTCAGTCTTTATAG	0.1		
16S rDNA	PS13	GGAGGCAGCAGTGGGGAATA	0.05	1,062	
	PS14	TGACGGGCGGTGTGTACAAG	0.05		
*tcdA*3'-end deletions	NK9	CCACCAGCTGCAGCCATA	0.17	700/1200	(33)
	NKV11	TGATGCTAATAATGAATCTAAAATGGTAAC	0.17		
*tcdC*	tcdC-F (-17)	AAAAGGGAGATTGTATTATGTTTTC	0.5	475	(34)
	tcdC-R (+462)	CAATAACTTGAATAACCTTACCTTCA	0.5		

### Multilocus sequencing typing

Seven *C. difficile* housekeeping genes were amplified according to the primers given on the official Multi-locus Sequencing Typing (MLST) website (http://pubMLST.org/cdifficile) using the method recommended by Griffiths et al. (29), followed by gene sequencing. The primers for MLST amplification and sequencing seen in Table 2. The sequencing results were submitted to the official website for comparison to obtain the allele type and the *C. difficile* sequence type (ST).

**Table 2:** The primers for MLST amplification and sequencing.

**Gene**	**Primer**	**Primer sequence**	**Gene length**	**References**
*adk*	adk1F	TTACTTGGACCTCCAGGTGC	635	(29)
	adk1R	TTTCCACTTCCTAAGGCTGC		
*atpA*	atpA1F	TGATGATTTAAGTAAACAAGCTG	674	
	atpA1R	AATCATGAGTGAAGTCTTCTCC		
*dxr*	dxr3F	GCTACTTTCCATTCTATCTG	525	
	dxr4R	CCAACTCTTTGTGCTATAAA		
*glyA*	glyA1F	ATAGCTGATGAGGTTGGAGC	625	
	glyA1R	TTCTAGCCTTAGATTCTTCATC		
*recA*	recA2F	CAGTAATGAAATTGGGAGAAGC	705	
	recA2R	ATTCAGCTTGCTTAAATGGTG		
*sodA*	sodA5F	CCAGTTGTCAATGTATTCATTTC	585	
	sodA6R	ATAACTTCATTTGCTTTTACACC		
*tpi*	tpi2F	ATGAGAAAACCTATAATTGCAG	640	
	tpi2R	TTGAAGGTTTAACACTTCCACC		

### PCR-ribotyping

PCR-ribotyping was performed according to nucleotide sequence polymorphisms in the 16S-23S rRNA intergenic spacer region of *C. difficile*. The gene fragments in this region were amplified according to the method recommended by Indra et al. (35). The size and peak of the fragments read by Gene Marker were submitted to the WEBRIBO database (http://webribo.ages.at) to determine the RT of *C. difficile*. Novel RTs were named with “LW” plus two Arabic numbers, such as LW01.

### Drug susceptibility test

The antimicrobial susceptibility test of the isolated *C. difficile* was performed using the agar dilution method recommended by the US Clinical and Laboratory Standards Institute (CLSI) M11-A8 (36). The following eight antibiotics were included in this study: vancomycin, clindamycin, erythromycin, levofloxacin, moxifloxacin, ciprofloxacin, meropenem, and metronidazole (Yuan ye Biotech., Shanghai, China). The results were interpreted with reference to the susceptibility breakpoints for anti-anaerobic drugs in CLSI 2017 version M100-S27 (37). Furthermore, for antibiotics with no susceptibility breakpoints in the CLSI document, the susceptibility breakpoints were proposed according to Huang's report (16).

### Statistical analysis

Statistical significance was determined using the chi-square test and univariate logistic regression in SPSS Statistics (version 22; IBM Corporation). The level of significance was set at *P* < 0.05 and *P* < 0.01.

The genetic relationships of the isolates was determined by cluster analysis using the minimum-spanning tree available in the BioNumerics software V6.5 (Applied Maths).

## Results

### Prevalence of CDI in dogs and cats

A total of 247 clinical samples were collected from five animal hospitals in Lanzhou City, of which dogs and cats accounted for 74.9% (185/247) and 25.1% (62/247), respectively. Among them, 10.3% (19/185) of dogs and 8.1% (5/62) of cats were positive for CDI. Five animal hospitals tested positive of CDI at rates of A (16.8%, 20/119), B (3.0%, 1/33), C (0%, 0/10), D (4.3%, 2/46) and E (2.6%, 1/39). Notably, in hospital A, 48.2% (119/247) of the samples were collected, but the positive samples account for 83.3% (20/24) of the total positive samples. The positive rate of hospital A was nearly 5 times higher than other hospitals. Treatment information and the health status of the dogs and cats were collected for statistical analysis (Table 3). Male and female dogs tested positive for CDI at rates of 10.5 and 10.0%, respectively. But male and female cats tested positive at rates of 3.6 and 11.7%, respectively. However, no significant difference in positive rates was observed in dogs and cats of different ages. Moreover, there was no significant correlation between diarrhea and isolated *C. difficile*. The positive rates for CDI in infectious, dermatological, and surgical diseases was diagnosed in 16.7, 16.7, and 13.0% of dogs, respectively, higher than its internal diseases, parasitic diseases, and immunizations. The rate of CDI positivity for infectious diseases in cats was 22.2%, higher than its other diseases' rates. The CDI positivity rate for vaccination in dogs and cats were 2.3 and 0.0%, which were extremely low. However, the positive rates for CDI in 25 hospital self-raised animals from five different animal hospitals were 18.8% (3/16) for dogs and 22.2% (2/9) for cats.

**Table 3:** Clinical information of sampled dogs and cats.

		**Dog (*****n*** = **185)**			**Cat (*****n*** = **62)**		
**Type**	**Variable**	**Number samples (%)**	**P-samples to CDI (%) ^a^**	***P* ^b^**	**Number samples (%)**	***P*-samples to CDI (%)**	***P* ^b^**
Gender	Female	90 (48.6)	9 (10.0)	0.90	34 (54.8)	4 (11.7)	0.36
	Male	95 (51.4)	10 (10.5)		28 (45.2)	1 (3.6)	
Animal Hospital	A	88 (47.6)	15 (17.0)	0.06	31 (50.0)	5 (16.1)	<0.01
	B	23 (12.4)	1 (4.3)		10 (16.1)	0	
	C	5 (3.0)	0		5 (8.1)	0	
	D	35 (19.0)	2 (5.7)		11 (17.7)	0	
	E	34 (18.0)	1 (3.0)		5 (8.1)	0	
Age	0–4 month	33 (17.8)	3 (9.1)	0.35	12 (19.4)	0	0.25
	5–12 month	43 (23.2)	3 (7.0)		24 (38.7)	1 (4.2)	
	13–72 month	73 (39.5)	11 (15.1)		24 (38.7)	4 (16.7)	
	≥73 month	36 (19.5)	2 (5.6)		2 (3.2)	0	
Diarrhea	Yes	55 (29.7)	8 (14.5)	0.21	15 (24.2)	2 (13.3)	0.59
	No	130 (70.3)	11 (8.5)		47 (75.8)	3 (6.4)	
Diseased animals	Interal disease	36 (28.8)	3 (8.3)	0.67	19 (47.5)	1(5.3)	<0.01
	Surgical disease	46 (36.8)	6 (13.0)		11 (27.5)	0	
	Skin disease	6 (4.8)	1 (16.7)		1 (2.5)	0	
	Infectious disease	30 (24.0)	5(16.7)		9 (22.5)	2(22.2)	
	Parasitic disease	7 (5.6)	0		0	0	
Healthy Animals	Vaccination ^c^	44 (73.3)	1 (2.3)	0.054	13 (59.0)	0	0.16
	Hospital self-raised animals ^d^	16 (26.7)	3 (18.8)		9 (41.0)	2(22.2)	

a Positive cases of C. difficile;

b univariate logistic regression;

c dogs and cats receive vaccine in the animal hospital;

d Hospital self-raised animals (such as foster pets, sheltered pets, pets raised by hospital employees, etc).

### Toxin genotyping and *TcdC* gene test

**Figure 1:**
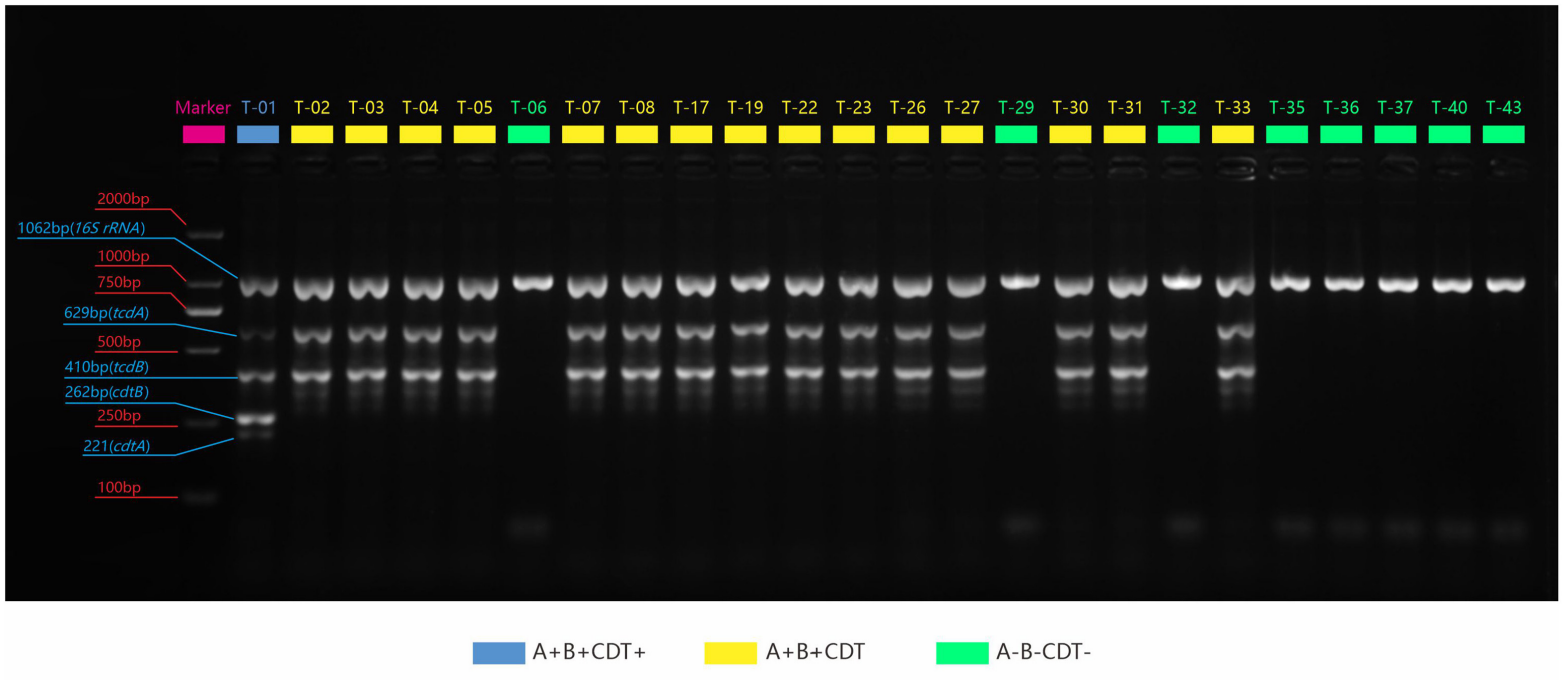
PCR amplification plots of the three toxin genes and the 16S rRNA genes. Blue: The strain contains four genes: *tcdA, tcdB, cdtA*, and *cdtB*; Yellow: It contains two genes: *tcdA, tcdB*; Green: It does not contain any toxin genes.

**Figure 2:**
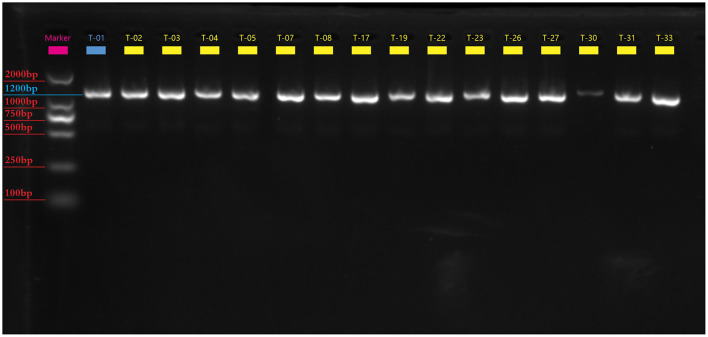
The electrophotogram of the *tcdA* gene PCR amplification. There are 16 toxin-producing Clostridium difficile *tcdA* gene bands at 1200bp.

We identified successfully 24 *C. difficile* strains by MALDI-TOF MS. There were 16 toxin-producing strains and 8 non-toxin-producing strains among them, with a toxic rate of 57.9% (11/19) in dogs and 100% (5/5) in cats. Five-fold PCR was used to amplify three toxin types: A+B+CDT+, A+B+CDT-, and A-B-CDT- (Figure 1). One of 16 toxic strains was A+B+CDT+ and other 15 were A+B+CDT-. Eight non-toxic strains all belonged to the A-B-CDT-. Next, the *tcdA* gene of 16 toxic strains was then amplified. Gel electrophoresis displayed typical *tcdA* bands at 1200 bp, meaning no gene deletion at 3' terminus of *tcdA* of all the 16 isolates (Figure 2). In other words, all the 16 isolates were normal toxic without toxic enhancement caused by gene deletion at 3' terminus of *tcdA*.

**Figure 3:**
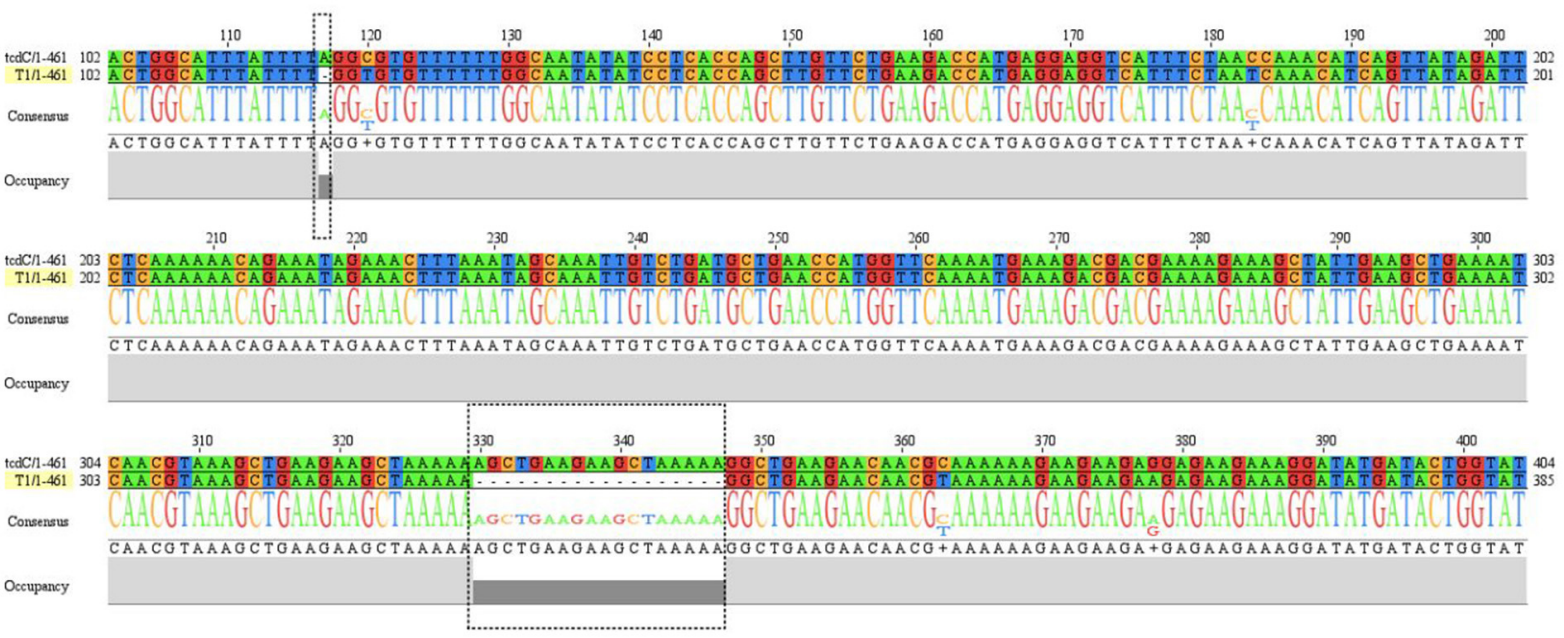
The sequence alignment between T1 strain (OP615994) and reference *tcdC* gene (NC_009089.1). *tcdC* gene of strain T1 has nucleotides deletions at position 117 and position 330-347 compared to reference sequence.

The *tcdC* gene of 16 strains were amplified and sequenced. The obtained sequences were compared to the standard *tcdC* sequences (NC_009089.1) from NCBI. Only one strain (OP615994) had a single nucleotide deletion at position 117 and 18 nucleotide deletions at positions 330–348 in the *tcdC* gene (Figure 3).

### MLST

Ten ST types were identified from seven housekeeping genes of 24 *C. difficile* strains in 3 MLST clades by MLST database matching. Among them, ST2, ST3, ST42, ST15, ST26, ST76 and ST133 strains were located in MLST clade1. ST1 strain and ST39 strain were located in MLST clade2 and MLST clade 4, respectively. The most common strains in the isolated *C. difficile* were ST42 (41.7%, 10/24), ST39 (16.7%, 4/24), and ST2 (12.5%, 3/24). The remaining seven STs included ST1, ST3, ST15, ST26, ST76, ST133 and the newly identified strain ST-UN, all of which had only one strain with an isolation rate of <5% (Table 4).

**Table 4:** Multilocus sequence type (MLST), ribotype, and toxin genotypes of 24 *clostridium difficile* clinical isolates.

**MLST clade**	**MLST ST**	**Ribotype**	**Toxin gene**	**NO. of isolates**
1	ST2	LW01	A+B+CDT-	1
	ST2	RT014	A+B+CDT-	2
	ST3	RT001	A+B+CDT-	1
	ST15	LW03	A-B-CDT-	1
	ST26	LW02	A-B-CDT-	1
	ST42	RT106	A+B+CDT-	9
	ST42	RT106	A-B-CDT-	1
	ST76	LW05	A-B-CDT-	1
	ST133	LW04	A+B+CDT-	1
2	ST1	RT027	A+B+CDT+	1
4	ST39	LW06	A-B-CDT-	4
-	STUN	LW04	A+B+CDT-	1

### RT

The data from 10 RTs were submitted to the WEBRIBO database for comparison. The RTs named RT027, RT014, RT106, and RT001 matched successfully with 14 strains. In addition, six new RTs were identified and named as LW “01–06”. Among these RTs, the most common type was RT106 (10/24), followed by LW06 (4/24) and RT014 (2/24) (Table 4).

### The relationship between MLST, RT, and the toxin gene

A+B+CDT+ toxic strain ST1 (RT027) was identified. The strain belonged to MLST clade 2, with a single nucleotide deletion at position 117 and 18 nucleotide deletions in the *tcdC* gene from positions 330–348 (Figure 3). This strain is highly virulent and has caused many large-scale outbreaks globally (38).

**Figure 4:**
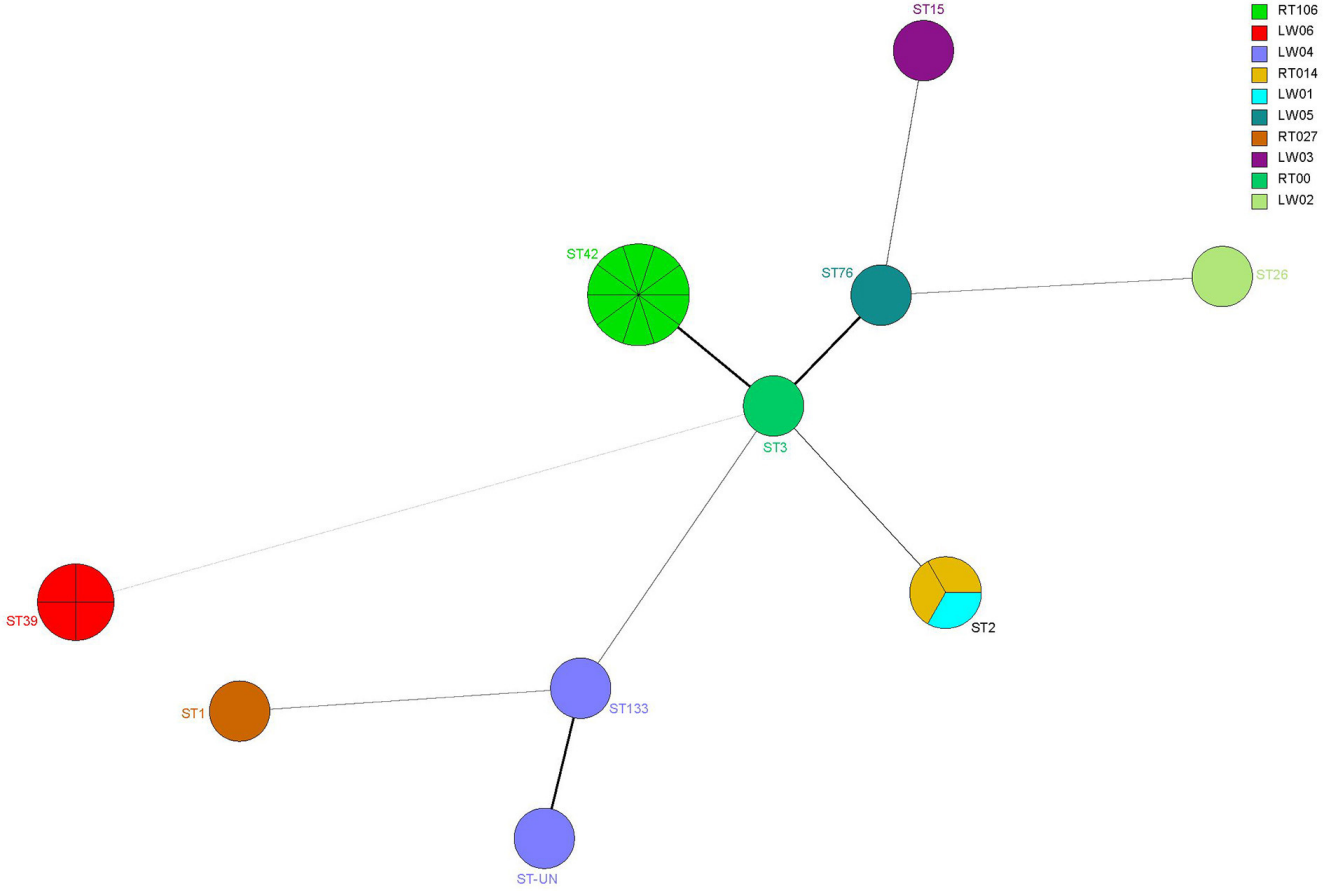
Analysis of the MLST minimum spanning tree for 24 *C. difficile* strains. Each circle represents a ST type, while the number next to the circle represents the ST type. The size of the circle represents the number of isolated strains; The colors represent different ribotypes; Kinship is shown by the lines connecting the circles.

**Figure 5:**
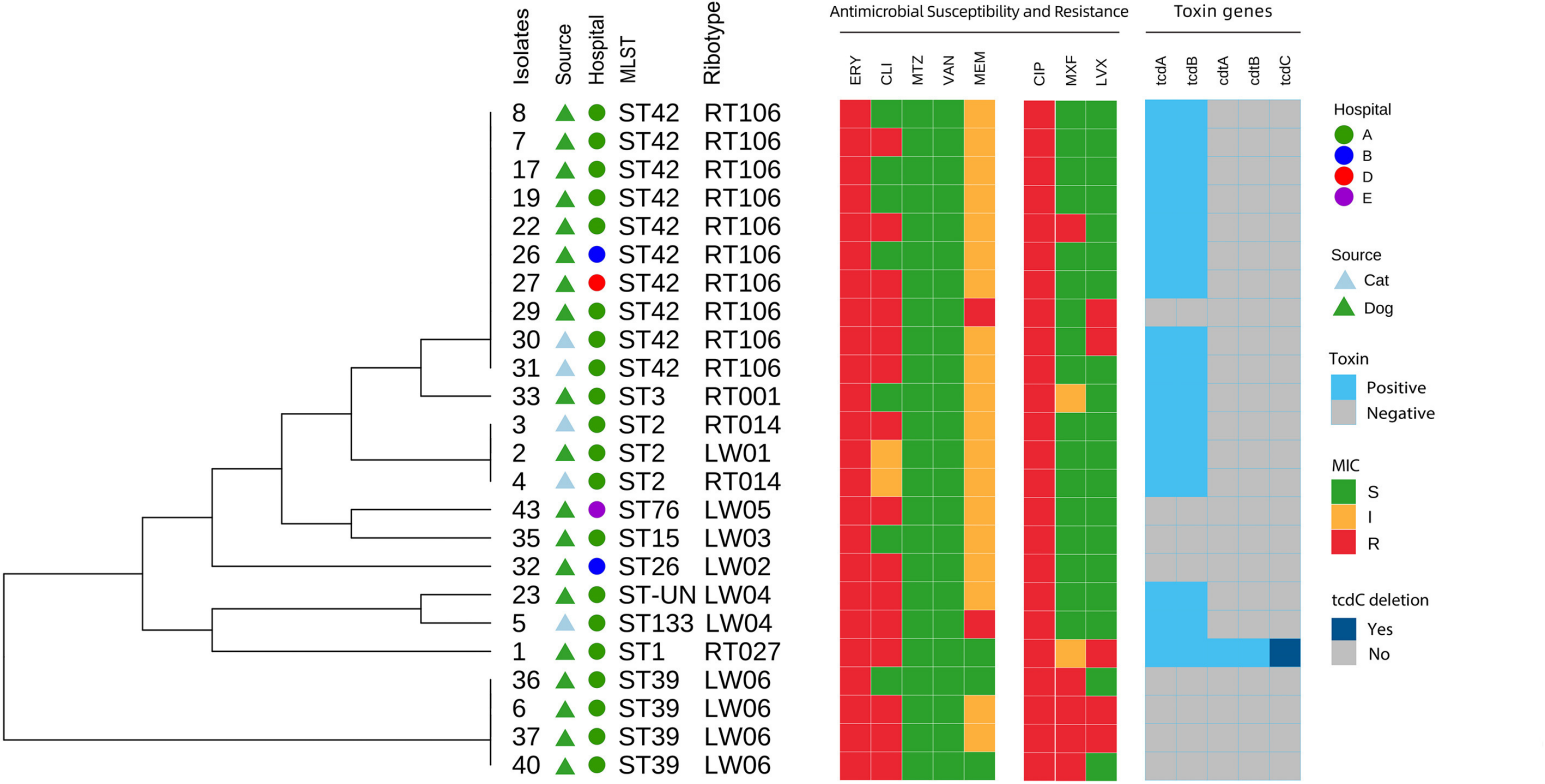
Core genome-based phylogenetic tree and distribution of *C. difficile* source, hospitals, toxin genes, MLST, ribotypes, antimicrobial susceptibility and resistance among isolates from dogs and cats.

ST42 (RT106), ST2 (RT014), ST2 (LW01), ST133 (LW04), ST-UN (LW04), and ST3 (RT001) were all found in 15 A+B+CDT-strains and belonged to the MLST clade 1 and were closely related (Figures 4, 5). ST3 and ST42 had a strong relationship. ST133 and ST-UN were similar and shared the same RT LW04 (Figure 4). Furthermore, the 355th adenine of ST133's housekeeping gene *atpA* was mutated to guanine. It led to the change in the *atpA* gene's allele from 1–4, thus forming a new mutant strain ST-UN.

Eight A-B-CDT- strains included ST39 (LW06), ST26 (LW06), ST76 (LW05), ST15 (LW03) and ST42 (RT106). ST42, ST76, ST15, and ST26 were members of MLST clade 1 and were closely related (Figures 4, 5). ST39 belonged to MLST clade 4. These five STs also had different RTs. In addition, nine strains of ST42 (RT106) were toxin-producing, while one was non-toxin-producing (Table 4 and Figure 5).

### Antimicrobial susceptibility analysis

All 24 *C. difficile* strains were susceptible to vancomycin and metronidazole but were resistant to erythromycin and ciprofloxacin. Clindamycin resistance was also high (62.5%). The resistance rates for levofloxacin, moxifloxacin, and meropenem were 20.8, 16.7, and 8.3%, respectively. The 50% minimum inhibitory concentrations (MIC_50_) of vancomycin and metronidazole were 0.5 and 4 μg/mL, respectively (Table 5).

**Table 5:** Antibiotic resistance rates and minimum inhibitory concentration (MIC) ranges for the 24 C. *diffificile* clinical isolates.

**Antimicrobial agent**	**All strains (n**+**24)**				**A**+**B**+ **strains (*****n*** = **16)**				**A-B- strains (*****n*** = **8)**			
	**MIC_50_**	**MIC_90_**	**Range**	**%R**	**MIC_50_**	**MIC_90_**	**Range**	**%R**	**MIC_50_**	**MIC_90_**	**Range**	**%R**	***P*-value**
	**(μg/mL)**	**(μg/mL)**	**(μg/mL)**		**(μg/mL)**	**(μg/mL)**	**(μg/mL)**		**(μg/mL)**	**(μg/mL)**	**(μg/mL)**		
Erythromycin	128	128	64–128	100	128	128	64–128	100	128	128	128–128	100	>0.05
Ciprofloxacin	128	128	16–128	100	128	128	16–128	100	64	128	16–128	100	>0.05
Metronidazole	4	4	≤4	0	4	4	≤4	0	4	4	≤4	0	>0.05
Clindamycin	32	32	2–256	62.5	8	32	2–32	56.2	32	256	2–256	75	>0.05
Meropenem	8	8	2–16	8.3	8	8	2–16	6.2	8	16	2–16	12.5	>0.05
Moxifloxacin	2	8	1–16	16.7	2	4	1–16	6.2	2	16	1–16	37.5	>0.05
Vancomycin	0.5	0.5	≤0.5	0	0.5	0.5	≤0.5	0	0.5	0.5	≤0.5	0	>0.05
Levofloxacin	2	64	1–64	20.8	2	16	1–64	12.5	1	64	1–64	37.5	>0.05

P-Value for resistant A +B+ strains versus A–B–strains.

**Figure 6:**
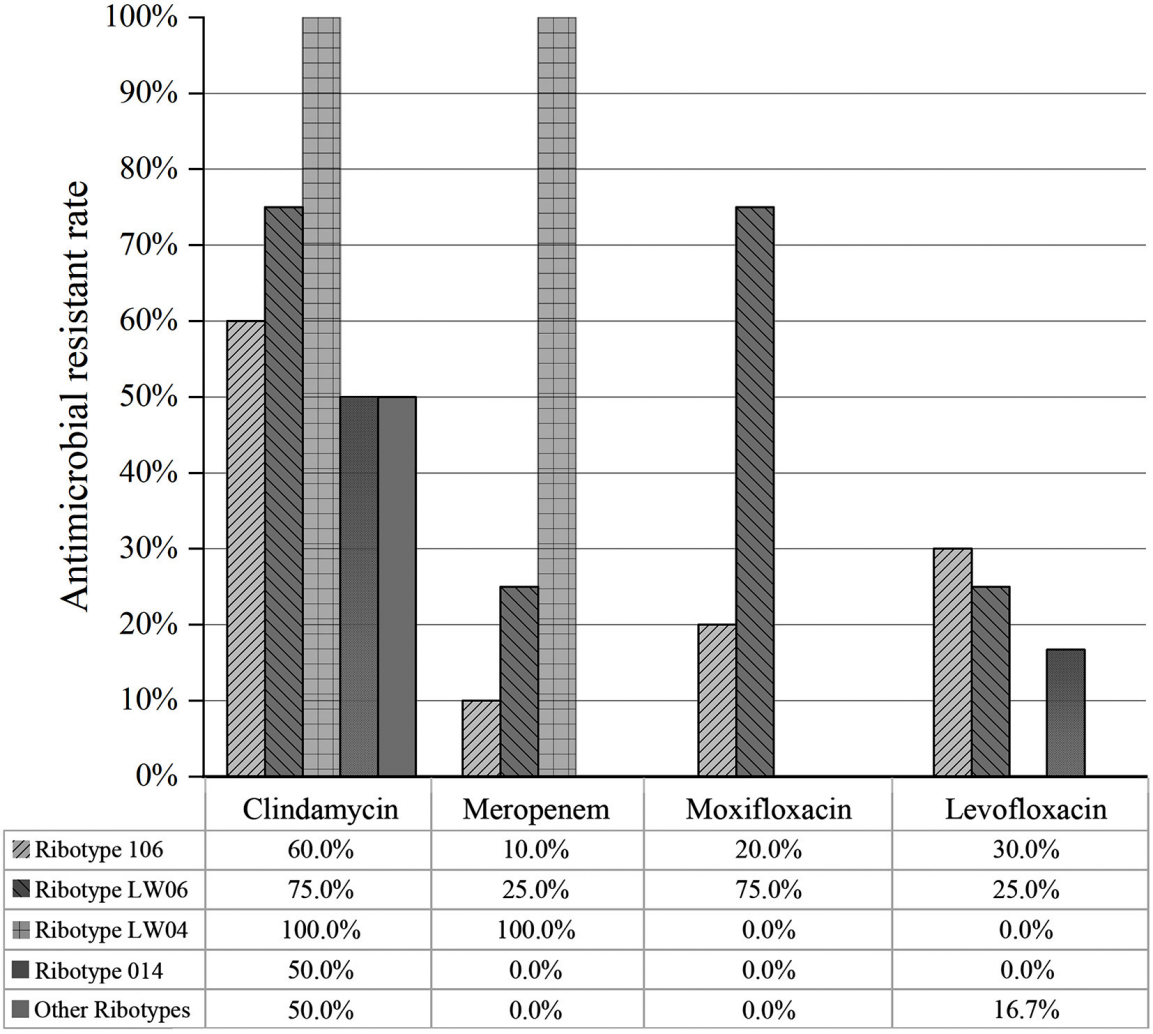
Drug resistance of different PCR ribotypes of 24 *C. difficile* isolates to antibiotics (clindamycin, meropenem, moxifloxacin and levofloxacin).

The resistance rates of 16 toxin-producing strains to clindamycin, levofloxacin, moxifloxacin, and meropenem were 56.2, 12.5, 6.2, and 6.2%, respectively. For eight non-toxin-producing strains, the resistance rates of clindamycin, moxifloxacin and levofloxacin and meropenem were 75, 37.5, 37.5, and 12.5%, respectively. The resistance of non-toxic strains was slightly higher than that of the toxic strains. The resistance rates of 10 ST42 (RT106) strains to clindamycin, meropenem, levofloxacin, and moxifloxacin were 60, 10, 30, and 20%, respectively. The resistance rates of the above antibiotics for four ST39 (LW06) strains were 75, 25, 25, and 75%, respectively. Moreover, two ST2 (RT014) strains exhibited 50.0% resistance to clindamycin but were susceptive to meropenem, moxifloxacin, and levofloxacin. Among above three common genotypes, ST42 (RT106) exhibited the highest resistance rate to levofloxacin and ST39 (LW06) were resistant to clindamycin and moxifloxacin (Figure 6).

## Discussion

*C. difficile* is an obligate anaerobe and needs specific sampling and isolation methods. In this study, samples were transported in the Amies transport medium to reduce the possibility of bacterial death due to oxygen exposure. Cefoxitin and cycloserine in the CCFA selective medium effectively inhibited the growth of other bacteria but did not affect *C. difficile*. In addition, sodium taurocholate in CCFA promoted the germination of *C. difficile* spores (39). As a result, the CCFA medium outperformed the traditional medium for *C. difficile* isolation. Currently, the principal method for identifying bacteria is 16S rRNA sequencing, which is labor-intensive and time-consuming. Therefore, the MALDI*-*TOF-MS identification method was applied in this study, which is rapid, high-throughput, and accurate (40).

*C. difficile* isolation rates in companion dogs and cats in Lanzhou city of China were 10.3% and 8.1%, respectively. The isolation rates were higher than those reported recently in China by Wei et al. (dogs 0.7% and cats 7%) (41). The CDI positive rates of dogs and cats from hospital A were significantly higher than other four hospitals (*P* < 0.01). The possible reasons for this for this difference were not determined as this was not the focus of the study. However, it possible that hospital A served as a potential source of C. difficile for patients or samples. This result was consistent to the molecular typing result that the prevalent strain in hospital A was RT106 (ST42). It is also possible higher CDI positivity was found in hospital A due to external conditions such as the geographic location, community socioeconomic conditions, animal population differences, or types of problems treated. On the other hand, the isolation rate of CDI from pets was less relative to pet status including age, diarrhea, and health condition (*P* > 0.05). The similar results were also observed from CDI epidemiologic study in dogs and cats in Madrid in Spain (20).

*C. difficile* is classified into different toxin types according to the type of toxin gene it carries. A+B+CDT- is the most commonly reported toxin type among toxic *C. difficile* strains, accounting for 70–90% of the total (42, 43). The toxin type with the highest toxin-producing capacity and prevalence is A+B+CDT+, such as RT027 and RT078, which caused CDI outbreaks worldwide (44). Furthermore, the clinical A-B+CDT- type is common in Chinese inpatients (45). The presence of only the *tcdB* gene may increase virulence due to its strong regulatory capacity (46). Therefore, the toxicity of A-B+CDT- toxin type cannot be ignored. There was also an identified *C. difficile* strain that only expressed the binary toxin CDT and CDT could alone cause CDI recurrence (27). However, there is currently no information on A+B-CDT- type of *C. difficile*.

In this study, bacterial toxin genes were amplified using five-fold PCR technology. There were 16 toxin-producing and 8 non-toxin-producing strains identified with toxin production rates of 57.9% in dogs (11/19) and 100% in cats (5/5). In North America, 50–73% and 7.1–34.8% of the toxin-producing *C. difficile* strains were isolated from dogs and cats, respectively (47–50). In Europe, toxic type isolation rates for dogs and cats were 5.5–58.0% and 5.6–80%, respectively (51–54). The isolation rate of toxin-producing strains in dogs in this study was similar to those in Europe and the United States, but the isolation rate in cats was higher. We isolated a *C. difficile* strain carrying the t*cdA, tcdB*, and CDT genes. *C. difficile* strains carrying the binary toxin usually leads to a high risk of morbidity and mortality and were of worldwide concern (8, 55).

A total of 10 STs and 10 RTs were identified in this study. Among them, ST42 (RT106) and ST2 (RT014/LW01) were the most common isolates, accounting for 41.7 and 12.5% of all isolates, respectively. ST39 (LW06), ST1 (RT027), and the remaining eight STs were located in MLST clade4, clade2 and clade1, respectively. STs from the same MLST clade were more closely related. The percentages of ST42 (RT106) and ST2 (RT014/LW01) among 16 toxin-producing strains were the highest. However, ST3 (RT001), ST1 (RT027), ST133 (LW04), and ST-UN (LW04) all had only one strain. ST42 (RT106) was the most common genotype. Recent reports showed that the most common RTs were RT014 and RT010 in dogs and RT014 in cats (56–58). RT106 was mainly found in some studies conducted in Brazil and Germany, and the isolation rate was relatively low (56). In Europe, RT014 is the most common cause of CDI-related diarrhea in human (13, 59). Although RT014 rarely causes outbreaks of CDI, it is highly adaptive and widespread in animals (60). In Berlin of Germany, the isolation rate of RT014 *C. difficile* in companion animals is 22.2% (4/18) (56). Two strains of RT014 were identified among 24 *C. difficile* strains in this study, with a relatively low isolation rate of 8.3%. Moreover, a RT027 strain of A+B+CDT+ was isolated, which possessed high toxin-producing capacity due to the binary toxin genes *cdtA* and *cdtB*. This strain had a single nucleotide deletion at site 117 and a nucleotide sequence deletion at sites 330–347 of the *tcdC* gene. The tcdC gene plays a negative regulatory role, and its deletion can increase the expression of toxin A and toxin B, thus enhancing the strain's virulence (61). Isolation of RT027 strain from companion animals was rarely reported worldwide. In Canada, a RT027 strain from a healthy dog in an animal hospital was firstly reported and then another report from dog in 2018 (56, 62). This study is the first report of the isolation of the RT027 strain in China on a healthy dog from an animal hospital. Hence, some highly virulent strains with high prevalence and pathogenicity in human may exist in animal hospitals, which increases the risk of cross-transmission between pets and human beings. In the future, enhancing inspection, prevention, and control are needed to avoid outbreaks of highly virulent *C. difficile* strains.

Eight non-toxin-producing *C. difficile* strains were isolated in this study and their STs were ST39, ST15, ST26, ST42 and ST76. Among them, ST39 (*n* = 4) was the most abundant, whereas the rest had only one strain. Moreover, we identified one non-toxin-producing ST42 strain among 10 strains. This same ST strain with both toxin-producing and non-toxin-producing properties exists in the PUBMLST database, but such a case is relatively rare.

The drug susceptibility analysis suggested that 24 C. *difficile* strains isolated in this study were susceptible to metronidazole and vancomycin with MIC_90_ of 4 and 0.5 μg/mL, respectively. Almost all C. *difficile* were susceptible to metronidazole and vancomycin. Therefore, they are currently the preferred antibiotics for the clinical treatment of CDI in human. Metronidazole is more commonly used in dogs than vancomycin for treating acute diarrhea and chronic enteritis (63, 64). Twenty-four C. *difficile* strains were resistant to erythromycin (100%) and ciprofloxacin (100%), which were similar to erythromycin (13–100%), ciprofloxacin (99%), clindamycin (55%), and moxifloxacin (34%) (65). C. *difficile* isolated from animals in China had a resistance rate of 93.7% to ciprofloxacin (66). The resistance rate of *C. difficile* to ciprofloxacin ranged between 80 and 100% in Shanghai of China (16). *C. difficile* also produced complete resistance to erythromycin. Although erythromycin was used less frequently in Spanish companion animals, resistance is relatively high (67). The resistance rate of 345 toxin-producing C. *difficile* strains to erythromycin was 69.5% in China (66). The resistance rates of toxin-producing C. *difficile* isolates to clindamycin and levofloxacin were 62.5 and 20.8%, respectively. The main MLST types of resistant isolates were ST42 (RT106), ST39 (LW06) and ST1 (RT027). All four strains of ST39 (LW06) developed antibiotic resistance. Therefore, ST39 (LW06) had high resistance to other antibiotics, which may be attributed to the different resistance rates among various regions and prevalent strains.

## Conclusion

The positive rates of C. *difficile* for dogs and cats in Lanzhou city of China were 10.3% (19/185) and 8.1% (5/62), respectively. The main pandemic strains were ST42 (RT106) and ST2 (RT014/LW01), which were also frequently reported in human-related studies. All isolated C. *difficile* strains were susceptible to metronidazole and vancomycin, while the resistant rates to erythromycin, ciprofloxacin, clindamycin, levofloxacin, moxifloxacin, and meropenem were 100, 100, 62.5, 20.8, 16.7, and 8.3%, respectively. The CDI of companion animals in China should be paid more attention to ensure animal welfare and public health security.

## Data availability statement

The datasets presented in this study can be found in online repositories. The name of the repository and accession numbers can be found below: NCBI; OP503451-OP503473.

## Ethics statement

The animal study was reviewed and approved by the Lanzhou Institute of Husbandry and Pharmaceutical Sciences of CAAS.

## Author contributions

X-WL and J-YL conceived of, proposed the idea, and designed the study. G-LW, S-HL, ZQ, L-XB, and W-BG conducted the experiments. J-YL and G-LW performed the acquisition and analysis of data. J-YL, G-LW, and X-WL wrote the manuscript. All authors contributed to the article and approved the submitted version.

## Funding

This research was supported by grants from the National Key R&D Program of China (2021YFD1800900), Natural Science Foundation of Gansu Province (22JR5RA042) and Science-technology innovation engineering of CAAS (25-LZIHPS-02).

## Conflict of interest

The authors declare that the research was conducted in the absence of any commercial or financial relationships that could be construed as a potential conflict of interest.

## Publisher's note

All claims expressed in this article are solely those of the authors and do not necessarily represent those of their affiliated organizations, or those of the publisher, the editors and the reviewers. Any product that may be evaluated in this article, or claim that may be made by its manufacturer, is not guaranteed or endorsed by the publisher.
